# Production networks and resilience: How dense production networks shield economies in financial crisis

**DOI:** 10.1371/journal.pone.0302012

**Published:** 2024-04-17

**Authors:** Petre Caraiani, Alina Mihaela Dima, Cristian Păun, Tănase Stamule, Madalina Vanesa Vargas

**Affiliations:** 1 Departament of Business Administration in foreign languages, Bucharest University of Economic Studies, Bucharest, Romania; 2 Institute for Economic Forecasting, Romanian Academy, Bucharest, Romania; 3 Department of International Relations, Bucharest University of Economic Studies, Bucharest, Romania; University of Education, PAKISTAN

## Abstract

The research delves into the underexplored area of how production network structures influence the severity of economic downturns, particularly during the last financial crisis. Utilizing the RSTAN database from the OECD, we meticulously derived critical measures from the input-output matrices for 61 economies. Our methodology entailed a panel analysis spanning from 2008 to 2010, which is a period marked by significant recessionary pressures. This analysis aimed to correlate economic performance with various production network metrics, taking into account control factors such as interest rates and the prevalence of service sectors. The findings reveal a noteworthy positive correlation between the density of production networks and economic resilience during the crisis, which remained consistent across multiple model specifications. Conversely, as anticipated, higher interest rates were linked to poorer economic performance, highlighting the critical interplay between monetary policy and economic outcomes during periods of financial instability. Given these insights, we propose a policy recommendation emphasizing the strategic enhancement of production network density as a potential buffer against economic downturns. This approach suggests that policymakers should consider the structural aspects of production networks in designing economic stability and growth strategies, thus potentially mitigating the impacts of future financial crises.

## 1. Introduction

The world has experienced many economic and financial crises in the last decades, such as the 2007–2008 Global Financial Crises (GFC). While there is ample literature on a large number of topics related to the GFC, this is the first attempt to study its impact from the perspective of production networks using a sample of consistent number of countries.

This article is developing a quantitative approach to the crisis from the perspective of production networks. The main research question is: *did the production network structure matter for the severity of the recession during and in the immediate aftermath of the GFC*?

There are several reasons that motivate our approach. Firstly, the present world economy is portrayed by high degrees of globalization, with firms sending out intermediary or finished products around the world and sourcing contributions from providers situated in various countries. This is now known as a global production network. The global economic crisis from 2009 pushed the topic of international trade into the scientific debate. The decline was dramatic as the exports fell suddenly back to the level of 2005. There are opinions saying that the international trade got back to normal once the crisis was over. Generally, the demand for goods is lowering during crises, diminishing the exports, but during upturns the companies will start exporting and expand their businesses again. The research challenge is whether the global networks are more strongly affected in case of a new crisis and what the recovery process implies. Our approach relies on domestic national network production. However, the spillovers among the production networks in different economies are implicitly present. Recent research has underlined that production networks are a potential key factor in economic growth [1] (Acemoglu and Azar, 2020). Further results also underscore the role of production networks in international trade, as found by [2] Dhyne et al. (2021). Last but not least, many recent studies outline the key role of production networks in amplifying the impact of aggregate shocks [3] (Farhi and Baqaae, 2018) for supply shocks [4] (Caraiani, 2022) for oil shocks, or for monetary policy shocks [5] (Caraiani, 2023). Taken together, our findings suggest that data on and modelling of domestic production networks are essential to understand the role and behavior of different types of firms in international trade.

Secondly, networks are omnipresent in all spheres of society and economy. Companies depend on information and communication networks and they try to create supply chains with many nodes to benefit from competitive advantages. If the network is large enough, it can bring various benefits to the involved actors. Nonetheless, before starting to benefit from the network, one must add value and maintain a linkage to the other associates. “Each node obtains an externality produced by the sum of knowledge in neighbour nodes” [6] (Matveenko. Et al., 2017). The inner equilibrium of the network is unique and represents time and effort actions from each node. The similarities between “its structure in terms of the types of nodes, and in networks with similar types structure agents in node” [6] (Matveenko. Et al., 2017) is the key to the stability of the network. Huremovic and Vega-Redondo (2016) [7] consider that, at equilibrium, “the profitability of any given firm is proportional to its network centrality, as given by a suitable generalization of the well-known measure of centrality proposed by Bonacich”.

Thirdly, the higher the production capabilities of a firm, the greater market shares it achieves. However, the input costs will be higher and number of consumers will decrease. Subsequently, low sales are a result of high production capability firms or, if the input prices are low, high sales result. Bernard et al. (2019) [8] suggest that “the production network accounts for more than half of firm size dispersion.” En masse, firms with higher attributes establish their failure or success, but models with just one wellspring of firm heterogeneity neglect to capture the heterogeneity in firms’ size.

From a methodological point of view, this paper combines network analysis with (panel) regression. The approach is justified, on one hand, by the need to characterize production network, and this can be done through the use of network measures derived from production networks. On the other hand, we also need to relate recession measures (like decrease of GDP) to these network measures and we can do it through panel regression. Panel regression is justified by the use of data for several countries.

Our paper has several relevant contributions in the field. Firstly, we approach the topic of the intensity of the financial crisis from a network production perspective. While most other studies focusing on the role of productions network in the transmission of shocks were based rather on single country data, mostly for the United States, we use a consistent dataset of 61 countries. Secondly, we selected the determinants of recessions by using several determinant factors, constructed using production networks. Thirdly, we analyze the role of a number of production-network related variables (like density, skewness, sector dominance), while controlling for different variables that considered significant in the recent literature (e.g. interest rate or the share of services).

## 2. Literature review

This part of the research is dedicated to review the most recent research regarding production networks and their role in modeling the macroeconomy or the financial sectors. There is a consensus that the application of network approach is quite diverse, signaling the increasing significance of this approach in macroeconomics. There are also other studies that researched the idea of input-output structure usage on the overall macroeconomic field.

Although the network approach to macroeconomics is rather old, with early applications exploiting the use of input-output production matrix, recently the interest in using networks in macroeconomic modelling has been reignited (Carvalho, 2014 [9] or Carvalho and Tahbaz-Salehi, 2019 [10]).

In a reference paper, Acemoglu et al. (2012) [11] showed that, when the intersectoral input-output links are taken into account, shocks at firm level (idiosyncratic shocks, in their terminology) can lead to aggregate fluctuations.

This research has also been extended at international level by considering global value chains–Antras et al. (2012) [12] measured the global chains of productions using specific measures like upstreamness (later, one also proposed the downstreamness as a complementary measure).

More recent work dealt with the transmission of shocks in network economics. Ozdagli and Weber (2017) [13] studied the idea of monetary policy, where the weights are derived from the input-output tables. Further work has been done by Caraiani (2019) [14] in the context of oil shocks, by Farhi et al. (2020) [15] for supply shocks, and by Caraiani et al. (2020) [16] or Caraiani (2023) [5] for the case of monetary policy shocks. These papers also exploit the structure of production networks, with the former focusing on measures like density and skewness and the latter using measures like upstreamness or downstreamness.

However, the applications of (production) networks is much more diverse. The role of infrastructure on production network and firm performance is directly analyzed by Bernard et al. (2019). Their research confirmed that an improved infrastructure that reduces “search costs and buyer-seller inefficiencies allow firms to match with more and better suppliers” and indirectly lowers the marginal costs of production. This means that firms that have a better geographical or network position have lower marginal costs and can produce more.

The recovery process of 2008 events generated important lessons and a lot of economists tried to develop various quantitative models related to the prevention of such disasters or early warning systems for future crises (EWS) such as Gao at al, (2013) [17]. The EWS models in the literature have a wide range of approaches. Gao at al. (2013) [17] list the works of Kaminsky, Lizondo, & Reinhart [18] who used the ‘*leading indicator approach*’; Bussiere & Fratzscher (2002) and Maddala (1989) [19, 20] referred to the ‘*discrete-dependent-variable approach based on logit*’; Goldberger (1972) [21] used ‘*probit models structural equation model*’, and ‘*network-based models*’ are used by Niemiraa & Saaty (2004) [22] and Ozkan-Gunay & Ozkan (2007) [23].

Some papers expanded the idea of networks in restoring the equilibrium in the Asian continent after the crises. While Thorbecke & Bhattacharyay (2012) [24] demonstrated that production networks have contributed to trade development and economic growth in East Asia, Ando (2013) [25] analysed machinery sectors in Japan concluding that ‘*regardless of whether creating demand shock or supply shock*, *the economic/natural disasters revealed the stability and robustness of production networks’*. Obashi (2009) [26] discussed the resiliency of international production networks in Japan underlying ‘*its stability after considering adverse effects of the crisis’*.

Some of the recent research also investigated how disproportionate the effect of a financial constraint is, when it affects only a small fraction of firms in the economy. For example, when one firm of the production networks meets financial constraints, it starts to invest less, purchases fewer intermediate goods, reduces its output and in the end the price of the good increases. The next firm in the chain will buy less from its supplier. In the end, each node of the chain will suffer from one’s financial constraint. As a result, “the aggregate effect of the financial shock is amplified as it propagates along the supply chain” (Campello et al., 2010) [27]. The question is if a future crisis can be caused by financial constraints of more nodes of the production networks simultaneously and how amplified the financial shocks of the input-output linkages are. As a response, Bigio and La’O (2016) [28] proposed a model to test the multiplier generated by the network effects and “predicts a range for the network liquidity multiplier of 1.8 to 6.3 during the Great Recession”.

At the same time, in spite of the rapidly growing number of studies in this field, the literature has not yet addressed the issue whether the structure of production networks matters for the severity of financial crises. This is an important question of interest for academics or decision makers. Better knowledge of the role of production network properties during crises can improve both our understanding and the policy design in such situations. Our paper aims at filling this gap.

## 3. Data and methodology

### 3.1. The visual flow chart

**Fig 1:**
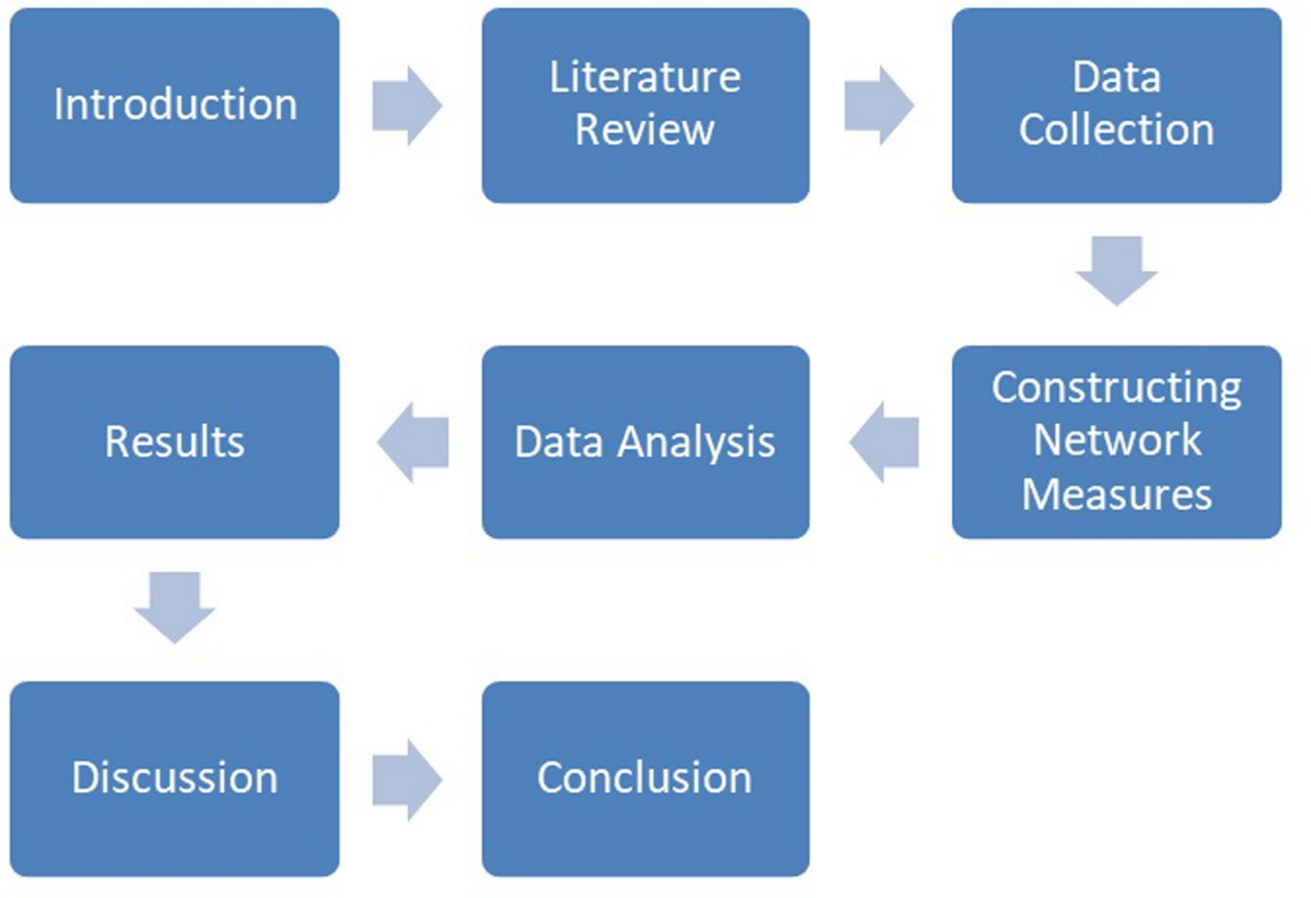
Visual flowchart of the paper. Source: Own analysis.

Following the research steps in this paper, the Fig 1 presents the visual flow chart for this paper.

### 3.2. Constructing the network measures

Since the network measures are relatively known in the specific literature, their utility is considered for this particular topic. The papers of Acemoglu et al. (2012) [11] or Miranda-Pinto (2021) [29] are relevant for the scientific literature in the field.

For the present research, the data on input-output structures of the production in a given economy are used for empirical analysis. This structure can be thought of and characterized by a weighted adjacency matrix. This matrix would represent a graph characterized by a number of *N* sectors and having a potential of *N*^*2*^ links (total number of links possible). We can denote this input-output matrix by *Ω*, with each element belonging to denoted by *ω*_*i*, *j*_. Each such element indicates the importance of intermediate inputs from a sector *i* to another sector *j*’s total expenditures on intermediates.

Following Acemoglu et al. (2012, 2016) [11, 30] we can capture each sector’s supplier importance by the Leontief inverse elements. An element j of the vector with the sectors’ supplier importance can be written as:

$$LL_{j}=\left(1^{\prime }{\left(I-(1-\alpha )1^{\prime }x\Omega^{\prime }\right)}^{-1}\right)j=\sum_{k=1}^{\infty }{\left((1-\alpha )1^{\prime }x\Omega^{\prime }\right)}_{j}^{\prime k}$$

Here *LL*_*j*_ is the *j*-th element of the vector LL, which is the vector with the sectors’ supplier importance, *1* stands for the vector of ones, while *x* indicates element-wise multiplication. The parameter *α* characterizes how important labor is in a certain sector, while *k* depicts higher order effects (for example, if *k = 1*, then we only have a direct effect of shocks of sector *j* to the sectors using the output of sector* j* as an input; however, for the case for *k>*1, the shocks affecting a sector *j* will affect the sectors that are linked indirectly to sector *j* too).

The Leontief matrix *L* can be further used to measure the asymmetry in the network (as given by the relative importance of the sectors in an economy). Following Acemoglu et al. (2016) [30], we can use sector dominance (hereafter denoted by DOM) to measure this asymmetry. First we can measure the influence of a sector by υ_t_:

$$v_{t}=\sum_{\left\{i=1\right\}}^{N}\beta_{i}L_{ij}$$

Here, the parameter *β*_j_ stands for the share of households’ consumption in sector *j* goods. Furthermore, *L*_*ij*_ is the element (*i*, *j*) in the Leontief matrix. Then, the sector dominance can be defined as:

$$DOM=\frac{v_{max}\sqrt{N}}{{\left(\sum_{i=1}v_{i}^{2}\right)}^{\left\{\frac{1}{2}\right\}}}$$

We also used the intensity with which intermediate inputs are used within sectors. To measure the intensity of the use of an intermediate input in total sales, we employed the weighted in-degree measured as:

$$d_{j}^{in}=\sum_{i}^{N}\left(1-\alpha_{j}\right)\omega_{ij}$$

For each specific country in the sample, we also measured the asymmetry by skewness of sectoral in-degree, denoted by *skewin*, and skewness of sectoral outdegrees, which is denoted by *skewout*.

Finally, another measure that is widely used in the literature is the density of a network, showing how well connected a network is. In this particular case where the total number of sectors is *N*, the total number of connections is *N(N-1)*, while the number of input-output connections is denoted by *T*, the density (*dens*, hereafter) is given by:

$$density\;=\frac{T-N}{N(N-1)}$$

We used different thresholds in order to count the number of connections, namely we used a threshold of 0.05% and a threshold of 0.1%.

### 3.3. Econometric framework

The baseline regression in the following specification is based on the recent papers by Miranda-Pinto (2021) [29], and also loosely on Acemoglu et al. (2015) [31]:

$$\Delta y_{t,j}=\beta_{0}+\beta_{1}*\;dens_{t,j}+\beta_{2}*\;skewin_{t,j}+\beta_{3}*\;skewout_{t,j}+\beta_{3}*x_{t,j}+\varepsilon_{t,j}$$

Here is the growth rate of real GDP in each year *t* of the sample, for country *j*. Since the data on the properties of networks is available only at annual frequencies, we used only data on annual GDP growth. A number of explanatory variables were introduced and discussed in section 3.2, and they are used to characterize the input-output production matrix for each country, namely the density of the I_O network in each year *t* (denoted by *dens*_*t*, *j*_) and for each country *j*, the skewness degree (we use two variables to characterize it, in-skewness, *skewin*_*t*, *j*_, as well as out-skewness, *skewout*_*t*, *j*_).

Furthermore, *x*_*t*, *j*_ stands for the control variables at time *t* and for country *j*. We included in the set of control variable the interest rate or the importance of services.

The baseline regression analysis without the control variables is the first step of the analysis; the second step is the inclusion of additional network properties, by considering the sector dominance index according to Acemoglu et al. (2012) [11], denoted by DOM, or the average use of intermediate inputs in the economy, AVMATSH. As a sensitivity analysis, we also vary the density measures by considering different degrees of density.

The next step of the analysis is the study of the impact of controlling for various macroeconomic and financial characteristics, including the share of services or the level of interest rate. Although we initially considered a larger set of control variables, given the limited data availability, we settled on the use of services share as well as of interest rates.

Firstly, the use of the interest rate is correlated with the importance of the monetary policy, which plays a key role in managing crises. During the GFC, some countries implemented aggressive monetary policies with the interest rate reaching the zero lower bound, while other were less restrictive in this area.

Miranda-Pinto (2021) [29] showed that, in the context of production network economies countries with larger service, shares are less volatile, and this explains the use of services as another variable in the analysis.

### 3.4. Data

We used several datasets to conduct the analysis. On one hand, we used the input-output structure for 61 economies as available in OECD Structural Analysis Database (STAN, hereafter) database. We chose the input-output matrices for the years 2008 to 2010, since the maximum impact of the crisis for most economies happened during this period of time.

Using this input-output matrix, several key measures of interest, including the density, the skewness, sector dominance or the average use of intermediate inputs (the details are given in Section 3.2 of the methodology above) were computed.

Since our focus is on the importance of the production network on the depth of the recession, we included in our analysis data on the real annual GDP growth for the years 2008 to 2010. The data source for these series is Penn World Table.

On the other hand, we also used a measure of short term interest rate from the OECD statistical database, where available. However, for the countries for which such data was not available at OECD, we used data from International Financial Statistics (IFS), International Monetary Fund, namely the monetary policy related interest rate.

The data sources for each of the series used are presented in Table 1, while Table 2 presents key descriptive statistics.

**Table 1:** Data sources.

Variable	Computation	Source
Density	Own computations	OECD STAN
In-degrees skewness	Own computations	OECD STAN
Out-degrees skewness	Own computations	OECD STAN
Sector Dominance	Own computations	OECD STAN
Average use of intermediate inputs	Own computations	OECD STAN
Interest rate	Raw data	OECD and IMF Financial Statistics
GDP growth rate	Own computations	Penn World Table
Services share	Own computations	OECD

Source: Author’s own contribution.

**Table 2:** Descriptive statistics.

Variable	Mean	Standard Deviation	Minimum	Maximum
Density 1	0.73	0.11	0.33	0.92
Density 2	0.63	0.11	0.28	8.82
Density 3	0.34	0.06	0.14	0.46
In-degrees skewness	-0.10	0.52	-1.32	1.27
Out-degrees skewness	2.00	0.76	0.43	4.75
Sector Dominance	1.28	1.04	1.15	1.53
Average use of intermediate inputs	0.38	0.05	0.21	0.47
Interest rate	4.02	3.25	0.13	15.82
GDP growth rate	0.38	2.00	-6.96	6.16

Source: Authors’ own contribution.

## 4. Findings

As previously explained, we used the growth rate as the dependent variable, which indicated the growth (or, in most cases, the recession) during the peak years of the crisis. In its baseline specification, the panel model is also balanced. However, when using the interest rate, since there is no data for several countries, the panel model is unbalanced. Table 3 shows the results of the baseline regression. We considered 61 countries (see S4 Appendix for the list of countries) with 3 yearly observations for each country (for the years 2008, 2009 and 2010) covering the peak of the crisis (for some countries, i.e. peripheral European economies, the crisis has been prolonged or aggravated due to the sovereign debt crisis).

**Table 3:** Baseline results.

Dependent Variable: GDP				
Method: Panel Least Squares				
Sample: 2008 2010				
Periods included: 3				
Cross-sections included: 61				
Total panel (balanced) observations: 183				
Variable	Coefficient	Std. Error	t-Statistic	Prob.
C	-10.92232	6.359646	-1.717441	0.0885
DENS_2	18.54579	9.697873	1.912356	0.0582
SKEWDIN	1.154379	0.904008	1.276956	0.2041
SKEWDOUT	-0.155789	1.162888	-0.133967	0.8937
	Effects Specification			
Cross-section fixed (dummy variables)				
R-squared	0.412279	Mean dependent var		0.387622
Adjusted R-squared	0.101133	S.D. dependent var		2.004751
S.E. of regression	1.900677	Akaike info criterion		4.391388
Sum squared resid	429.8960	Schwarz criterion		5.513831
Log likelihood	-337.8120	Hannan-Quinn criter.		4.846369
F-statistic	1.325034	Durbin-Watson stat		3.907687
Prob(F-statistic)	0.094616			

We tested for random effects (see S3 Appendix), and the results indicate that the hypothesis of random effects is rejected on the basis of the Hausman test. Furthermore, tests for redundant fixed effects indicated that the fixed effects are not redundant (see S2 Appendix). Given these findings, the subsequent results are based on the fixed-effects approach.

To take into account the sensitivity of results to the density measure used, we also employ a different measure of density. Again, the results indicated that the density of an economy had a positive impact on economic growth, as shown in Table A.1 in S1 Appendix. In other words, denser economies tended to perform better during the Great Recession.

Generally, the results here underscore again what has been repeatedly been found for other types of shocks (oil, like in Caraiani (2019) [14], supply as in Farhi et al. (2020) [15] or monetary policy shocks as in Caraiani (2023) [5]: production networks generally have a significant role in modeling the impact of aggregate shocks. Depending on the network measure used, they can amplify or smoothen the impact of these shocks.

### 4.1. Robustness of results

We also performed a set of additional analyses by considering various alternative measures of input-output production networks, like average use of intermediate inputs, AVMATSH, or DOM, a measure of sector dominance (Tables A.2 and A.3 in S1 Appendix). The density continues to have a positive role, while AVMATSH had a negative impact on economic growth during this period (in other words, it worsened the recessions).

In Table A.4 in S1 Appendix, the role of services is estimated. The literature, for instance Miranda-Pinto (2021) [29], indicates a positive role of services in reducing volatility across different countries. Here, the impact of services is rather negative. This might also come from the fact that the services sector also covers financial and banking activities, which had a significant role during the last financial crisis. In other words, countries with more developed financial markets tended to have a more negative impact on their GDP in the last crisis.

We finally analyzed the role of the interest rate in Table A.5 in S1 Appendix. As expected, the interest rate had a negative role on the rate of growth during the crisis, thus underlining its known role of smoothening the impact of the recessions (in other words, higher interest rates had a negative impact on economic growth, or made the recession worse. This effect also includes the quantitative easing measures that helped the most developed economies to counter the negative impact of the crisis). We also check for the role of services, which showed a negative impact, possibly reinforcing the suggested explanation above.

Overall, except the last regression, there is a key role for the density of the networks (that is, how well connected the sectors of an economy are), that not only is positive, but also robust following the various specifications (or when changing its definition).

## 5. Discussion of results and implications

### 5.1. Theoretical mechanisms

The evidence presented in this paper can serve as a background for both improving the understanding of the current generation of models, and for getting a more solid intuition about the mechanisms through which financial crises are transmitted. Recent research, for example Benguria and Taylor (2020) [32] found, using data on a large number of financial crises during the past 200 years, that financial crises are actually demand shocks. However, besides making a distinction between supply and demand shocks, financial crises are essentially adverse shocks that challenge the resilience of economies.

Thus, our research can be interpreted as suggesting that financial crises shocks transmit themselves more hardly within denser networks. To our knowledge, this result is newer and a summary discussion within the context of a theoretical model is worth doing. However, it is important to underline that the question is more difficult and should be studied in the future. The model follows the contribution by Miranda-Pinto (2021) [29] who extends the model by Acemoglu et al. (2012) [11] by considering CES technologies for production.

The model is a multi-sectoral economy with *N* sectors. Each sector has a number of *N* competitive firms. The constant returns to scale technology for a firm in a sector j is:

$$Q_{j}=Z_{j}{\left[\alpha_{j}^{\left\{\rho_{q}\right\}}L_{j}^{\left\{\frac{\epsilon_{Q,j}-1}{\epsilon_{Q,j}}\right\}}+{\left(1-\alpha_{j}\right)}^{\left\{\rho_{q}\right\}}L_{J}^{\left\{\frac{\epsilon_{Q,j}-1}{\epsilon_{Q,j}}\right\}}\right]}^{\frac{\epsilon_{Q,j}}{\epsilon_{Q,j}-1}}$$

There is a composite material bundle *M*_*j*_ which can be written as:

$$M_{j}={\left(\sum_{\left\{i=1\right\}}^{N}\omega_{\left\{ij\right\}}^{\left\{\rho_{M}\right\}}M_{\left\{ij\right\}}^{\frac{\epsilon_{M,j}-1}{\epsilon_{M,j}}}\right)}^{\frac{\epsilon_{M,j}}{\epsilon_{M,j}-1}}$$

We used *Q*_*j*_ to denote the output of the representative firm in sector *j*, *Z*_*j*_ stands for the total factor productivity, *L*_*j*_ is the labor, *M*_*j*_ is the material bundle used in sector j and *M*_*ij*_ is the amount of materials purchased in sector *j* from firms in sector *i*. For each firm in sector *j*, the labor share is denoted by *α*_*j*_. We also characterize the elasticity of substitution between labor and materials at sectoral level by *ε*_*Q*, *j*_. Furthermore, this specification also relies on the parameters *ρ*_*Q*_ and *ρ*_*M*_, which are costs of complexity parameters (also known as *love of variety*).

There is also a representative household that is characterized by the following utility function:

$$U(C,L)=\frac{C^{\left\{1-\zeta \right\}}-1}{1-\zeta }-\psi \frac{L^{\left\{1+\eta \right\}}}{1+\eta }$$

Here, *C* is the consumption bundle, *L* is the total labor supply, the parameter *ζ* is the income elasticity labor supply, while *η* is the inverse Frisch elasticity of labor supply. The household faces the following budget constraint:

$$wL+\sum_{\left\{j=1\right\}}^{N}\pi_{j}=P_{C}C$$

Where:

$$C=\sum_{\left\{j=1\right\}}^{N}{\left(\beta_{j}^{\left\{\frac{1}{\epsilon_{D}}\right\}}C_{j}^{\left\{\frac{\epsilon_{D}-1}{\epsilon_{D}}\right\}}\right)}^{\frac{\epsilon_{D,j}}{\epsilon_{D,j}-1}}$$

Here, *P*_*C*_ is the price index for the consumption bundle *C*. We also have that the consumption shares *β*_*j*_ satisfy the relationship:

$$\sum_{\left\{j=1\right\}}^{N}\beta_{j}=1$$

While Miranda-Pinto (2021) [29] provides theoretical evidence that the impact of productivity shocks is influenced by the density of the production network, a similar mechanism can be depicted intuitively here for demand shocks too (following the argument of Benguria and Taylor (2020) [32], financial crises can be seen as acting as demand shocks, and since in a neoclassical model, demand shocks show up in TFP movements, i.e. as productivity shocks, according to Bai et al. (2017) [33], the results in Miranda-Pinto (2021) [29] approximately hold).

The key mechanism acting here is the cost of complexity which operates only when sectoral technologies have non-unitary elasticity between labor and intermediates.

### 5.2. Relationship with the literature

This analysis focused on the relevance of the density of an economy on the economic growth, while also emphasizing the particular impact of the use of intermediate inputs, the role of services in reducing the volatility across different countries included in our sample and the influence of interest rate during crisis time. The paper found a consistent and positive impact for the density of the networks for economic growth confirming that the countries with denser structure of the economic system performed better during crisis time, confirmed also by Miranda-Pinto (2021) [29].

A consistent and negative impact of the life average use of intermediate inputs indicates that the countries with lower life average use of intermediate inputs performed better during crisis time. Furthermore, a negative impact of the importance of services on the GDP’s growth rate confirms that countries with GDP depending more on the services performed less during the crisis (the services sector, especially financial services, reduced the recovery of the countries depending more on them) and, finally, the interest rate negatively impacted the GDP growth rate during crisis time (higher interest rates reduced the economic recovery).

Our findings continue the analysis of previous studies confirming the importance of the networks for economic growth (Bernard et al. (2018) [34], Carvalho and Tahbaz-Salehi (2019) [10], or Carvalho (2014) [9]); the study also stresses the importance of financial constraints for economic recovery–consistent with the findings of Bigio and La’O (2016) [28]; the average life use of intermediate inputs is influencing the resilience of economies to the crisis, which is consistent with the findings of Obashi (2009) [26], Thorbecke and Bhattacharyay (2012) [24].

The results of this study are important for a better understanding of the economic growth during crisis time, for improving the capacity of economic recovery by reconsidering the role of the networks density and the dominance of economic sectors and for addressing public policies to prevent and to reduce the negative impact of crisis contagion effect that significantly increased as countries became more globalized and inter-connected.

The results also underline again what has been recently found for other aggregate shocks: production networks have a significant role in the transmission of aggregate shocks.

### 5.3. Limitations

There are several limitations of this empirical research: the reduced number of years (data covers only 3 years, determined by the focus on the crisis period and its immediate aftermath), the reduced number of countries (only 61 countries out of 180 countries, determined by lack of data on input-output tables for a larger number of countries). At the same time, this study is one of the first to extend the network analysis over a sample of such dimension (while focusing strictly on the maximum impact of the financial crisis).

A further limitation is the use of the linearity hypothesis (We thank a reviewer for pointing this out): for example the relationship between the density and the economic performance can be overturned in turbulent times, while the connectivity between enterprises can also be a source of adverse network risks. Several works in this direction are done to Helbing (2013) [35], Acemoglu et al. (2016) [30] or Demertzis (2020) [36].

Another issue considered relevant is the low-interest rate environment. Our results point to a negative impact of interest rates: the higher the interest rate, the lower the economic performance during and in the aftermath of the GFC. However, especially in the aftermath of the GFC, there has been a low-interest rate environment, which can drastically change (and it has changed since due to the increased inflation).

Finally, we also underscored that our focus on the domestic input-output tables is by no means less relevant(We thank another reviewer for underlining this limitation). Firstly, although there are some alternative data sources, the number of countries is much smaller. Secondly, while we acknowledged that there is a number of small open economies in the sample, where the business cycles are driven mostly by external variables, our sample includes generally well developed economies (OECD members), not to mention the G7 economies. Thirdly, we noticed that our goal was to understand how the structure of the domestic economy mattered for the impact of the GFC.

## Conclusions

The current paper enlarges the existent research regarding the relevance of the production network on the depth of the recession. Taking into consideration 61 economies from the OECD database, the analyzed data confirm previous findings that outline the importance of network structure in understanding macroeconomic dynamics.

The findings of this research are very important for understanding the effects of the business cycles (especially during recession time) on the structure of production and the existing networks created between market players. The market is the place that harmonizes different and apparently independent actions and resources allocation. Our findings confirmed that economies with higher complexity and more interconnected sectors and operators were less impacted by crisis and more resilient. The importance of the density of networks should be included in any economic and investment decision, and it should be reconsidered when public policies are addressed to the economic environment.

Due to its direct impact on the level of interest rates, the monetary policy should take into account to supporting the increase of the complexity/density of network. The focus should be more redirected to aggregate supply and its complexity and sophistication, including the density of the network between all intermediate and final goods suppliers. At the global level, the diversification of supply chains, their better inter-connectivity and strategic location will clearly benefit economic globalization.

Further improvements of the study could refine the way of measuring the economic density, the dominance of the sectors, and they could include a closer look to the financial constraints by introducing variables particularly referring to the importance of the financial services for GDP formation, financial sophistication and depth, the quality of financial services and institutions.

Our study can be extended in many ways. First, it could also consider clearer theoretical mechanisms for the transmission of the crisis. Second, it could additionally take into account the importance of international transactions. Last but not least, future work could benefit from extending the sample and by looking at a larger number of crises and/or recessions.

## Supporting information

S1 Appendix Contains Tables A.1-A.5. (DOCX)

S2 Appendix Further tests: Fixed effects vs random effects. (DOCX)

S3 Appendix Further tests: Hausman tests for random effects. (DOCX)

S4 Appendix The list of countries (in their order in the OECD RSTAN database). (DOCX)
