# Supplementary material for: Production networks and resilience: How dense production networks shield economies in financial crisis
Source: PLoS One. 2024 Apr 17;19(4):e0302012. doi: 10.1371/journal.pone.0302012 (PMC11023220; doi:10.1371/journal.pone.0302012)
Supplement: S1 Appendix — (DOCX) [file pone.0302012.s001.docx]

# Appendix A

Table A.1. Baseline Results – Controlling Different Density Measures

| Dependent Variable: GDP | | |  |  |
| --- | --- | --- | --- | --- |
| Method: Panel Least Squares | | |  |  |
| Sample: 2008 2010 | | |  |  |
| Periods included: 3 | | |  |  |
| Cross-sections included: 61 | | |  |  |
| Total panel (balanced) observations: 183 | | | |  |
|  |  |  |  |  |
|  |  |  |  |  |
| Variable | Coefficient | Std. Error | t-Statistic | Prob. |
|  |  |  |  |  |
|  |  |  |  |  |
| C | -10.11946 | 6.393638 | -1.582740 | 0.1161 |
| DENS_1 | 15.14494 | 8.574370 | 1.766303 | 0.0799 |
| SKEWDIN | 1.105451 | 0.903537 | 1.223470 | 0.2236 |
| SKEWDOUT | -0.219491 | 1.170357 | -0.187542 | 0.8516 |
|  |  |  |  |  |
|  |  |  |  |  |
|  | Effects Specification | |  |  |
|  |  |  |  |  |
|  |  |  |  |  |
| Cross-section fixed (dummy variables) | | | |  |
|  |  |  |  |  |
|  |  |  |  |  |
| R-squared | 0.409694 | Mean dependent var | | 0.387622 |
| Adjusted R-squared | 0.097178 | S.D. dependent var | | 2.004751 |
| S.E. of regression | 1.904853 | Akaike info criterion | | 4.395778 |
| Sum squared resid | 431.7874 | Schwarz criterion | | 5.518221 |
| Log likelihood | -338.2137 | Hannan-Quinn criter. | | 4.850759 |
| F-statistic | 1.310956 | Durbin-Watson stat | | 3.892224 |
| Prob(F-statistic) | 0.103233 |  |  |  |

Table A.2. Further Results – Controlling for the Average Use of Material Inputs

| Dependent Variable: GDP | | |  |  |
| --- | --- | --- | --- | --- |
| Method: Panel Least Squares | | |  |  |
| Sample: 2008 2010 | | |  |  |
| Periods included: 3 | | |  |  |
| Cross-sections included: 61 | | |  |  |
| Total panel (balanced) observations: 183 | | | |  |
|  |  |  |  |  |
|  |  |  |  |  |
| Variable | Coefficient | Std. Error | t-Statistic | Prob. |
|  |  |  |  |  |
|  |  |  |  |  |
| C | 2.988888 | 8.428484 | 0.354618 | 0.7235 |
| DENS_2 | 24.50487 | 9.806441 | 2.498854 | 0.0138 |
| SKEWDIN | 0.903232 | 0.891488 | 1.013174 | 0.3131 |
| SKEWDOUT | -0.679500 | 1.159052 | -0.586255 | 0.5588 |
| AVMATSH | -43.02484 | 17.55748 | -2.450514 | 0.0157 |
|  |  |  |  |  |
|  |  |  |  |  |
|  | Effects Specification | |  |  |
|  |  |  |  |  |
|  |  |  |  |  |
| Cross-section fixed (dummy variables) | | | |  |
|  |  |  |  |  |
|  |  |  |  |  |
| R-squared | 0.440740 | Mean dependent var | | 0.387622 |
| Adjusted R-squared | 0.137413 | S.D. dependent var | | 2.004751 |
| S.E. of regression | 1.861924 | Akaike info criterion | | 4.352679 |
| Sum squared resid | 409.0780 | Schwarz criterion | | 5.492661 |
| Log likelihood | -333.2702 | Hannan-Quinn criter. | | 4.814770 |
| F-statistic | 1.453017 | Durbin-Watson stat | | 3.858014 |
| Prob(F-statistic) | 0.040413 |  |  |  |
|  |  |  |  |  |

**Table A.3. Further Results – Controlling for the Sector Dominance**

| Dependent Variable: GDP | | |  |  |
| --- | --- | --- | --- | --- |
| Method: Panel Least Squares | | |  |  |
| Sample: 2008 2010 | | |  |  |
| Periods included: 3 | | |  |  |
| Cross-sections included: 61 | | |  |  |
| Total panel (balanced) observations: 183 | | | |  |
|  |  |  |  |  |
|  |  |  |  |  |
| Variable | Coefficient | Std. Error | t-Statistic | Prob. |
|  |  |  |  |  |
|  |  |  |  |  |
| C | -1.079486 | 10.34747 | -0.104324 | 0.9171 |
| DENS_1 | 15.14767 | 8.565984 | 1.768351 | 0.0796 |
| SKEWDIN | 1.876155 | 1.138628 | 1.647734 | 0.1021 |
| SKEWDOUT | -0.393630 | 1.179682 | -0.333675 | 0.7392 |
| DOM | -6.705204 | 6.038202 | -1.110464 | 0.2691 |
|  |  |  |  |  |
|  |  |  |  |  |
|  | Effects Specification | |  |  |
|  |  |  |  |  |
|  |  |  |  |  |
| Cross-section fixed (dummy variables) | | | |  |
|  |  |  |  |  |
|  |  |  |  |  |
| R-squared | 0.415799 | Mean dependent var | | 0.387622 |
| Adjusted R-squared | 0.098944 | S.D. dependent var | | 2.004751 |
| S.E. of regression | 1.902990 | Akaike info criterion | | 4.396311 |
| Sum squared resid | 427.3217 | Schwarz criterion | | 5.536292 |
| Log likelihood | -337.2624 | Hannan-Quinn criter. | | 4.858401 |
| F-statistic | 1.312268 | Durbin-Watson stat | | 3.900198 |
| Prob(F-statistic) | 0.101860 |  |  |  |
|  |  |  |  |  |
|  |  |  |  |  |

**Table A.4. Further Results – Controlling for The Share of Services**

| Dependent Variable: GDP | | |  |  |
| --- | --- | --- | --- | --- |
| Method: Panel Least Squares | | |  |  |
| Sample: 2008 2010 | | |  |  |
| Periods included: 3 | | |  |  |
| Cross-sections included: 61 | | |  |  |
| Total panel (balanced) observations: 183 | | | |  |
|  |  |  |  |  |
|  |  |  |  |  |
| Variable | Coefficient | Std. Error | t-Statistic | Prob. |
|  |  |  |  |  |
|  |  |  |  |  |
| C | 16.92309 | 8.015060 | 2.111412 | 0.0368 |
| DENS_2 | 16.12426 | 8.850716 | 1.821803 | 0.0710 |
| SKEWDIN | 0.474328 | 0.834840 | 0.568166 | 0.5710 |
| SKEWDOUT | 0.037361 | 1.060428 | 0.035232 | 0.9720 |
| SERVICES | -41.82265 | 8.315657 | -5.029387 | 0.0000 |
|  |  |  |  |  |
|  |  |  |  |  |
|  | Effects Specification | |  |  |
|  |  |  |  |  |
|  |  |  |  |  |
| Cross-section fixed (dummy variables) | | | |  |
|  |  |  |  |  |
|  |  |  |  |  |
| R-squared | 0.516025 | Mean dependent var | | 0.387622 |
| Adjusted R-squared | 0.253530 | S.D. dependent var | | 2.004751 |
| S.E. of regression | 1.732075 | Akaike info criterion | | 4.208098 |
| Sum squared resid | 354.0097 | Schwarz criterion | | 5.348079 |
| Log likelihood | -320.0409 | Hannan-Quinn criter. | | 4.670188 |
| F-statistic | 1.965849 | Durbin-Watson stat | | 3.301681 |
| Prob(F-statistic) | 0.000767 |  |  |  |
|  |  |  |  |  |
|  |  |  |  |  |

**Table A.5: Further Results – Controlling for the Interest Rate**

| Dependent Variable: GDP | | |  |  |
| --- | --- | --- | --- | --- |
| Method: Panel Least Squares | | |  |  |
| Sample: 2008 2010 | | |  |  |
| Periods included: 3 | | |  |  |
| Cross-sections included: 58 | | |  |  |
| Total panel (balanced) observations: 174 | | | |  |
|  |  |  |  |  |
|  |  |  |  |  |
| Variable | Coefficient | Std. Error | t-Statistic | Prob. |
|  |  |  |  |  |
|  |  |  |  |  |
| C | 33.21706 | 9.223330 | 3.601417 | 0.0005 |
| DENS_2 | 3.877978 | 9.463206 | 0.409795 | 0.6827 |
| SKEWDIN | 0.597129 | 0.858332 | 0.695685 | 0.4881 |
| SKEWDOUT | 1.081207 | 1.111736 | 0.972540 | 0.3329 |
| SERVICES | -56.35532 | 9.270937 | -6.078708 | 0.0000 |
| INTRATE | -0.192306 | 0.070365 | -2.732961 | 0.0073 |
|  |  |  |  |  |
|  |  |  |  |  |
|  | Effects Specification | |  |  |
|  |  |  |  |  |
|  |  |  |  |  |
| Cross-section fixed (dummy variables) | | | |  |
|  |  |  |  |  |
|  |  |  |  |  |
| R-squared | 0.562820 | Mean dependent var | | 0.356726 |
| Adjusted R-squared | 0.318629 | S.D. dependent var | | 2.011813 |
| S.E. of regression | 1.660656 | Akaike info criterion | | 4.126915 |
| Sum squared resid | 306.1133 | Schwarz criterion | | 5.270711 |
| Log likelihood | -296.0416 | Hannan-Quinn criter. | | 4.590909 |
| F-statistic | 2.304834 | Durbin-Watson stat | | 3.211442 |
| Prob(F-statistic) | 0.000064 |  |  |  |
|  |  |  |  |  |
