# Supplementary material for: Production networks and resilience: How dense production networks shield economies in financial crisis
Source: PLoS One. 2024 Apr 17;19(4):e0302012. doi: 10.1371/journal.pone.0302012 (PMC11023220; doi:10.1371/journal.pone.0302012)
Supplement: S2 Appendix — (DOCX) [file pone.0302012.s002.docx]

# Section B. Further tests: Fixed Effects vs Random Effects

**Table B.1. Baseline Regression**

|  |  |  |  |  |
| --- | --- | --- | --- | --- |
|  |  |  |  |  |
| Effects Test | | Statistic | d.f. | Prob. |
|  |  |  |  |  |
|  |  |  |  |  |
| Cross-section F | | 1.155885 | (60,119) | 0.2500 |
| Cross-section Chi-square | | 84.032656 | 60 | 0.0220 |

**Table B.2. Baseline Results – Controlling Different Density Measures**

|  |  |  |  |  |
| --- | --- | --- | --- | --- |
|  |  |  |  |  |
| Effects Test | | Statistic | d.f. | Prob. |
|  |  |  |  |  |
|  |  |  |  |  |
| Cross-section F | | 1.115892 | (60,119) | 0.3030 |
| Cross-section Chi-square | | 81.686311 | 60 | 0.0328 |
|  |  |  |  |  |
|  |  |  |  |  |

**Table B.3. Further Results – Controlling for the Average Use of Material Inputs**

|  |  |  |  |  |
| --- | --- | --- | --- | --- |
|  |  |  |  |  |
| Effects Test | | Statistic | d.f. | Prob. |
|  |  |  |  |  |
|  |  |  |  |  |
| Cross-section F | | 1.298389 | (60,118) | 0.1150 |
| Cross-section Chi-square | | 92.769428 | 60 | 0.0042 |
|  |  |  |  |  |
|  |  |  |  |  |

**Table B.4. Further Results – Controlling for the Sector Dominance**

|  |  |  |  |  |
| --- | --- | --- | --- | --- |
|  |  |  |  |  |
| Effects Test | | Statistic | d.f. | Prob. |
|  |  |  |  |  |
|  |  |  |  |  |
| Cross-section F | | 1.133217 | (60,118) | 0.2796 |
| Cross-section Chi-square | | 83.269468 | 60 | 0.0251 |
|  |  |  |  |  |
|  |  |  |  |  |

**Table B.5. Further Results – Controlling for the Share of Services**

|  |  |  |  |  |
| --- | --- | --- | --- | --- |
|  |  |  |  |  |
| Effects Test | | Statistic | d.f. | Prob. |
|  |  |  |  |  |
|  |  |  |  |  |
| Cross-section F | | 1.501779 | (60,118) | 0.0309 |
| Cross-section Chi-square | | 103.828060 | 60 | 0.0004 |
|  |  |  |  |  |
|  |  |  |  |  |

**Table B.6. Further Results – Controlling for the Interest Rate**

|  |  |  |  |  |
| --- | --- | --- | --- | --- |
|  |  |  |  |  |
| Effects Test | | Statistic | d.f. | Prob. |
|  |  |  |  |  |
|  |  |  |  |  |
| Cross-section F | | 1.572685 | (57,111) | 0.0215 |
| Cross-section Chi-square | | 103.007503 | 57 | 0.0002 |
|  |  |  |  |  |
|  |  |  |  |  |
