# Supplementary material for: Production networks and resilience: How dense production networks shield economies in financial crisis
Source: PLoS One. 2024 Apr 17;19(4):e0302012. doi: 10.1371/journal.pone.0302012 (PMC11023220; doi:10.1371/journal.pone.0302012)
Supplement: S3 Appendix — (DOCX) [file pone.0302012.s003.docx]

**Section C. Further tests: Hausman Tests for Random Effects**

**Table C.1. Baseline Regression**

|  |  |  |  |  |
| --- | --- | --- | --- | --- |
|  |  |  |  |  |
| Test Summary | | Chi-Sq. Statistic | Chi-Sq. d.f. | Prob. |
|  |  |  |  |  |
|  |  |  |  |  |
| Cross-section random | | 6.797576 | 3 | 0.0786 |
|  |  |  |  |  |
|  |  |  |  |  |

**Table C.2. Baseline Results – Controlling Different Density Measures**

| Correlated Random Effects - Hausman Test | | | |  |
| --- | --- | --- | --- | --- |
| Equation: EQ_GDP_BASELINE | | |  |  |
| Test cross-section random effects | | | |  |
|  |  |  |  |  |
|  |  |  |  |  |
| Test Summary | | Chi-Sq. Statistic | Chi-Sq. d.f. | Prob. |
|  |  |  |  |  |
|  |  |  |  |  |
| Cross-section random | | 6.290842 | 3 | 0.0983 |
|  |  |  |  |  |
|  |  |  |  |  |

**Table C.3. Further Results – Controlling for The Average Use of Material Inputs**

|  |  |  |  |  |
| --- | --- | --- | --- | --- |
|  |  |  |  |  |
| Test Summary | | Chi-Sq. Statistic | Chi-Sq. d.f. | Prob. |
|  |  |  |  |  |
|  |  |  |  |  |
| Cross-section random | | 13.950240 | 4 | 0.0075 |
|  |  |  |  |  |
|  |  |  |  |  |

**Table C.4. Further results – Controlling for the Sector Dominance**

|  |  |  |  |  |
| --- | --- | --- | --- | --- |
|  |  |  |  |  |
| Test Summary | | Chi-Sq. Statistic | Chi-Sq. d.f. | Prob. |
|  |  |  |  |  |
|  |  |  |  |  |
| Cross-section random | | 7.999037 | 4 | 0.0916 |

**Table C.5. Further Results – Controlling for the Share Of Services**

|  |  |  |  |  |
| --- | --- | --- | --- | --- |
|  |  |  |  |  |
| Test Summary | | Chi-Sq. Statistic | Chi-Sq. d.f. | Prob. |
|  |  |  |  |  |
|  |  |  |  |  |
| Cross-section random | | 25.949242 | 4 | 0.0000 |
|  |  |  |  |  |
|  |  |  |  |  |

**Table C.6. Further Results – Controlling for the Interest Rate**

|  |  |  |  |  |
| --- | --- | --- | --- | --- |
|  |  |  |  |  |
| Test Summary | | Chi-Sq. Statistic | Chi-Sq. d.f. | Prob. |
|  |  |  |  |  |
|  |  |  |  |  |
| Cross-section random | | 32.665256 | 5 | 0.0000 |
|  |  |  |  |  |
