# Supplementary material for: Production networks and resilience: How dense production networks shield economies in financial crisis
Source: PLoS One. 2024 Apr 17;19(4):e0302012. doi: 10.1371/journal.pone.0302012 (PMC11023220; doi:10.1371/journal.pone.0302012)
Supplement: S4 Appendix — (DOCX) [file pone.0302012.s004.docx]

# Appendix D. The List of Countries (in their order in the OECD RSTAN Database)

| Australia |
| --- |
| Austria |
| Belgium |
| Canada |
| Chile |
| Czech |
| Denmark |
| Estonia |
| Finland |
| France |
| Germany |
| Greece |
| Hungary |
| Iceland |
| Ireland |
| Israel |
| Italy |
| Japan |
| Korea |
| Latvia |
| Luxembourg |
| Netherlands |
| New Zealand |
| Norway |
| Poland |
| Portugal |
| Slovakia |
| Slovenia |
| Spain |
| Sweden |
| Switzerland |
| Turkey |
| UK |
| US |
| Argentina |
| Bulgaria |
| Brazil |
| Brunei |
| Colombia |
| Costa Rica |
| Cyprus |
| Cyprus |
| Cyprus |
| Hong Kong |
| Croatia |
| Indonesia |
| India |
| Cambodia |
| Lithuania |
| Malta |
| Malaysia |
| Morocco |
| Peru |
| Philippines |
| Romania |
| Russia |
| Saudi Arabia |
| Singapore |
| Thailand |
| Tunisia |
| Taiwan |
| Vietnam |
| South Africa |
